# Christian physicalism and the biblical argument for dualism

**DOI:** 10.1007/s11153-021-09811-0

**Published:** 2021-09-18

**Authors:** Ralph Stefan Weir

**Affiliations:** School of History and Heritage, University of Lincoln, Brayford Pool, Lincoln, LN6 7TS UK

**Keywords:** Christian physicalism, Dualism, The Bible, Soul, Body

## Abstract

This paper examines whether biblical descriptions of the intermediate state imply dualism of the sort that rules out physicalism. Certain passages in the Bible seem to describe persons or souls existing without their bodies in an intermediate state between death and resurrection. For this reason, these passages appear to imply a form of dualism. Some Christian physicalists have countered that the passages in question are in fact compatible with physicalism. For it is compatible with physicalism that, although we are necessarily constituted by physical bodies, we can continue to exist without our current bodies in the intermediate state by being constituted by replacement bodies. I argue that broadly Gricean considerations significantly weaken this response. In its place, I propose a new, linguistic objection to the biblical argument for dualism. The linguistic objection says that biblical descriptions of an intermediate state cannot imply dualism in the sense that contradicts physicalism because physicalism is defined by a concept of the physical derived from modern physics, and no term in the biblical languages expresses that concept. I argue that the linguistic objection is less vulnerable to Gricean considerations than the constitution objection. On the other hand the linguistic objection also makes concessions to dualism that some Christian physicalists will find unacceptable. And it may be possible to reinforce the biblical argument for dualism by appeal to recent research on ‘common-sense dualism’. The upshot for Christian physicalists who wish to remain open to the biblical case for an intermediate state is therefore partly good, partly bad. The prospects for a Biblical argument for dualism in the sense that contradicts physicalism are limited but remain open.

## Introduction

A number of biblical passages appear to describe human persons or souls existing without their bodies in an intermediate state between death and resurrection. It has been argued on this basis that the Bible implies a form of soul-body dualism. Some Christian physicalists have replied that the intermediate state, if there is one, is compatible with physicalism. For it is compatible with physicalism that human persons are only contingently constituted by their current bodies, and that they, or their ‘souls’, continue to exist in the intermediate state by being constituted by a replacement body. This paper takes the discussion forward in two ways. First, I introduce a distinct, linguistic objection to the biblical argument for dualism, construed as an argument against Christian physicalism: physicalism is standardly defined by a concept of the physical derived from modern physics; the biblical languages have no term that expresses this concept; for this reason, it seems that the linguistic resources of the Bible prevent it from affirming dualism in the sense that contradicts physicalism. Secondly, I describe a strategy for reinforcing the biblical argument for dualism by appealing to conversational implicature. I argue that this strategy significantly weakens the constitution objection but that it is not comparably effective against the linguistic objection. For Christian physicalists who wish to remain open to the biblical case for an intermediate state the upshot is partly good and partly bad. On the one hand, if the relevant passages imply an intermediate state at all, then it appears that the intermediate state must be a *nonbodily* state in the sense of ‘body’ found in these passages. On the other hand, it does not follow that the intermediate state is *nonphysical* in the sense that defines physicalism. So, although the Bible might imply dualism of some sort, it is much less clear that it implies dualism in the sense that contradicts physicalism. In the final section, I suggest that the biblical argument for dualism might be further reinforced by appeal to research on the ‘naturalness’ of thinking of human persons in a dualist way. However I give reasons for thinking that this strategy will have limited dialectical force pending further research. The aim of this paper is not to throw additional weight to one or other side of the debate about Christian physicalism, but to open two new lines of argument that should be of interest to all parties.

## Christian physicalism

It is generally recognised that most historical Christian thinkers from the church fathers down have advocated some form of soul-body dualism (cf. Rickabaugh, 2018b). An early exception is Hobbes (*Leviathan* 1.4.21; 1.12.7; 3.34.1–26; 3.38.4; 4.44.15–16). Hobbes rejects the view that the soul is an incorporeal substance on the basis that ‘substance’ and ‘body’ are synonyms, and that ‘incorporeal substance’ is therefore a contradiction. Hobbes also urges that, for several reasons, dualism does not fit well with Christianity. First, the term ‘soul’, in the Bible, does not refer to an immaterial substance, but to life or to a living creature. Secondly, the Bible teaches the resurrection of the body, not the immortality of the soul. Thirdly, belief in an immaterial soul among Christians can be traced to the influence of pre-Christian Greek culture. Hobbes’s contemporaries were largely unswayed. But twentieth-century theologians gave new life to the Hobbesian outlook. Important contributions to this development include Wheeler Robinson’s (1911) argument that dualism misrepresents the Old Testament view of human persons, and Oscar Cullmann’s (1958) argument that the New Testament teaches the resurrection of the body only, not the immortality of the soul. By the late twentieth century, opposition to dualism was commonplace among Christian theologians.

While the theological considerations that Hobbes advanced in favour of Christian materialism resemble those that gained favour among twentieth century theologians, Hobbes’s philosophical case for materialism has enjoyed no such afterlife. However, in the twentieth century philosophers developed a new line of argument in favour of a position that resembles Hobbes’s materialism insofar as it is a form of monism which leaves no room for nonphysical souls. This is the position known as ‘physicalism’. David Papineau (2007) sets out an influential account of the rise of physicalism. On Papineau’s account, with the progress of physical science it became clear that physical events in the brain are brought about by prior physical causes in a way that leaves no room for nonphysical souls (or minds, etc.) to exert a systematic causal influence on the brain. It is generally assumed that if nonphysical souls exist at all then they exert a systematic causal influence on the brain. And so it was concluded that nonphysical souls do not exist and that there is nothing more to humans than our physical bodies. This is known as the ‘causal exclusion argument’ for physicalism. In conjunction with various auxiliary arguments, the casual exclusion argument led to the establishment of physicalism as the standard position in philosophical anthropology. Whether or not Papineau’s story is historically or scientifically accurate, the causal exclusion argument remains the leading argument for physicalism in the recent philosophical literature.

The philosophical and theological developments of the twentieth century summarised here suggest, for independent reasons, that there is nothing more to human persons than our physical bodies. It is therefore natural that Christian philosophers have brought the two together to establish a tradition of Christian physicalism. Christian physicalism can be seen as combining the latest developments in theology and philosophy to yield a scientifically and theologically respectable account of human nature. I discuss a position advanced by two Christian physicalists, Lynne Rudder Baker and Kevin Corcoran, at some length in what follows. Other representatives of Christian physicalism include Peter van Inwagen (1995), Trenton Merricks (1999, 2001, 2009) and Nancy Murphy (2006).

Physicalism is often called ‘materialism’. This is because physicalism resembles the traditional materialism of Democritus, Epicurus and Hobbes, if nothing else, in that it is a monistic ontology that rules out nonphysical souls. But there is an important difference between present-day physicalism and traditional materialism, and hence between the Christian physicalism that has become popular of late and the Christian materialism of Hobbes. The classic materialists limited their ontologies to entities that satisfy the pretheoretical concept of bodies as things that take up space. Democritus, Epicurus and Hobbes sought to understand reality in terms of the motions of solid, extended objects of various shapes and sizes. Physicalists, by contrast, limit their ontology to the kind of things *described by physics*. (Jessica Wilson (2006, fn. 1) lists some examples.) This revision in our understanding of ‘materialism’ developed gradually over the course of the nineteenth and twentieth centuries, in response to the increasing tendency of physicists to describe reality in terms of entities such as overlapping fields, volumeless particles and wave-particle dualities, that do not fit comfortably within traditional or pretheoretical conceptions of matter. (At least one defence of what I am calling ‘Christian physicalism’, Corcoran (2018), argues in favour of the label ‘materialism’ rather than ‘physicalism’. But Corcoran uses the term ‘materialism’ to indicate that he endorses a nonreductive view about higher-level properties, not because he is committed to an old-fashioned conception of matter. On the contrary, Corcoran (2018, p. 374) defines ‘materialism’ in terms of the concept of ‘physical’ things where this refers to ‘the sorts of entities it is the job of physicists to investigate’.) For this reason, despite its resemblance to traditional materialism, physicalism is a distinctively modern position that could not have been formulated prior to the development of modern physics. In many contexts, the difference between modern, scientifically inspired physicalism and traditional materialism can be ignored, and it is usually left in the background in discussions of Christian physicalism. But as I explain in what follows, this difference makes room for a novel linguistic objection to the biblical argument for dualism, insofar as that argument is supposed to pose a threat to Christian physicalism.

## The biblical argument for dualism

Christian physicalism has obvious merits. But Christian physicalists must contend with the biblical argument for dualism. This argument is advanced in its most comprehensive form by John Cooper (2000, 2001, 2007, 2009a, 2009b, 2015, 2018). A similar line of argument was advanced earlier by Joseph Ratzinger (1988, 104–29). Several other scholars have added their support (e.g. Moreland, 2014, 40–73; Gundry, 2005; Esler, 2005; Steiner, 2015; Rickabaugh, 2018a, 2019). (It might be urged that there are really several distinct biblical arguments for dualism. If so, it is Cooper’s argument on which this paper focuses.) Cooper’s presentation of the argument is complex. But its main pillar is the claim that a number of biblical passages entail that human persons, or their souls, continue to exist in an intermediate state between death and resurrection. It is the continued existence of persons or souls in the intermediate state that Cooper regards as decisive (cf. Cooper, 2018, 532, 537). Cooper divides his examples into non-Pauline, and Pauline texts. A particularly clear non-Pauline example is Matthew 10:28 where Jesus tells his disciples not to fear ‘those who kill the body but cannot kill the soul’. A particularly clear Pauline example is 2 Corinthians 5:8 where Paul says that ‘we would rather be away from the body and at home with the Lord.’ (Both NRSV translations.) Cooper claims that the doctrine of the intermediate state, as it is expressed in these passages, implies a form of soul-body dualism.

Cooper reinforces this argument in two ways. First, he argues that, contrary to the allegation that dualism is a Greek import to Christianity, even the Old Testament contains passages that imply that persons or souls continue to exist in the absence of the body after death. These include descriptions of the departed in Sheol, like Isaiah 14:9–10 and the raising of the spirit of Samuel in 1 Samuel 28. Along the way, Cooper (2000, 53–4) also rebuts the argument, advanced by Hobbes, Robinson and others that the Old Testament terms translated as ‘soul’, ‘ר֫וּחַ’ and ‘נֶ֫פֶשׁ’ are not used to refer to disembodied persons after death. Cooper contends that the term ‘רְפָאִים’, usually translated as ‘shades’, *is* used in this way, and that the metaphysical implications are the same, irrespective of which term is used. Secondly, Cooper appeals to evidence that intertestamental Jews generally, and the Pharisees in particular, held that prior to resurrection, the dead are ‘alive somewhere, somehow, in an interim state’ (Wright, 2003, 130). This, Cooper argues, makes it natural to understand biblical affirmations of the resurrection in the same way, especially given that Jesus and Paul align themselves with the Pharisees on the question of the resurrection at Matthew 22:23–33 and Acts 23:6–9.

Some of the passages Cooper identifies, including Matthew 10:28, seem to imply that humans have ‘souls’ (usually ‘ψυχαί’) that exist in the intermediate state. Others, including 2 Corinthians 5:8, seem to entail that human persons themselves do so. Cooper assumes that for the Bible, as for Plato or Descartes, for a person’s soul to continue to exist following death *just is* for that person to continue to exist. But Cooper acknowledges that the passages that he identifies are also consistent with a trichotomist view on which a person and a soul are distinct things, *both of which* continue to exist after death. This possibility does not bear on the responses to Cooper’s argument discussed in this paper, and so I will follow Cooper in using ‘soul’ and ‘person’ interchangeably for the thing that, on his reading of the Bible, continues to exist in the intermediate state.

Cooper focuses on the thesis that the Bible implies some kind of soul-body dualism. He does not give close attention to whether this is dualism in the sense that contradicts physicalism. But this is clearly a matter of significance, given the important place of physicalism in recent philosophy. In what follows my principal concern is whether the passages Cooper identifies imply dualism in the sense that contradicts physicalism. In taking this approach, I do not mean to suggest that Cooper is committed to the thesis that the passages he identifies imply dualism in that sense. Rather, I view this as an issue that merits our attention in its own right. I am particularly interested in two philosophical objections to the biblical argument for dualism, considered as an argument against Christian physicalism, and in whether those objections can be surmounted. For this purpose, it is convenient to focus on Cooper’s central claim that New Testament passages like Matthew 10:28 and 2 Corinthians 5:8 imply that the persons or souls continue to exist without the body after death. And it is convenient to treat these two passages as representative. I adopt this approach because the philosophical questions that I discuss can be adequately represented in abstraction from the wider details of biblical scholarship, not because I think the wider scholarship irrelevant or that Matthew 10:28 and 2 Corinthians 5:8 carry most or all of the weight of the biblical argument for dualism.

## The constitution objection

One way in which Christian physicalists can respond to the biblical argument for dualism is by arguing that the biblical texts, taken together, do not entail that persons or souls continue to exist between death and resurrection as Cooper claims. This response has received detailed criticism, however (e.g. Cooper, 2018). It is not part of the purpose of this paper to evaluate that line of response. A second way in which Christian physicalists can respond to the biblical argument for dualism is by arguing that *even if* the passages identified by Cooper entail that persons or souls continue to exist in an intermediate state, this is compatible with physicalism. I focus on two responses of this second variety, one well-established, the other new. The principal advantage of these responses is that they allow Christian physicalists to remain open-minded about the biblical case for an intermediate state. I begin with a response pioneered by Baker (1995, 2004, 2011, 2018) and, following her, Corcoran (1998, 2002, 2005, 2006, 2018), which I will refer to as the ‘constitution objection’.

The constitution objection takes as its background a theory of the relationship between human persons and their bodies which Baker labels the ‘constitution view’ (Baker, 1995 490–96; 2000 *passim*; 2004, 331–5; 2011 47–52; 2018, 341–4). According to the constitution view, as Baker presents it, human persons stand in the same kind of relationship to their physical bodies as a statue does to a piece of bronze, a banknote to a piece of paper, or a flag to a piece of cloth. That is, human persons are not identical to their bodies, and we could in principle exist without our bodies. But human persons are necessarily constituted by *some* body or other. Baker and Corcoran have developed sophisticated characterisations of the constitution relations that they take to obtain between persons and their bodies. But the details need not detain us here. The important point is that it is plausible that there is some constitution relation such that the thesis that persons are wholly constituted by physical bodies is sufficient for physicalism without requiring that human persons depend necessarily on the existence of their *current* bodies. If so, the constitution view will yield a version of physicalism that is consistent with the claim that a human persons—or their ‘souls’, assuming that this comes to the same thing—will continue to exist without their bodies after death, so long as they do so in virtue of being constituted by a *replacement* body. As Baker (2004, 11) puts it, ‘for all that we know, persons in the intermediate state are constituted by intermediate-state bodies.’ The constitution view appears, therefore, to be straightforwardly consistent with passages like Matthew 10:28 and 2 Corinthians 5:8, even though it is a kind of physicalism. If so, it appears that Christian physicalists who accept the constitution view need not worry about the biblical argument for dualism.

In comparison with arguments that the Bible does not imply the existence of an intermediate state at all, the constitution objection has met with a sympathetic reception among proponents of the biblical argument for dualism. For example, in the preface to the second edition of his book, Cooper (2000, xxvii) himself concedes that the constitution objection shows that some kinds of physicalism are at least consistent with the passages he identifies as implying dualism. He describes this as ‘a significant development that may bear fruit in the monism-dualism debate’. Cooper continues to urge that a dualist reading is preferable. But he does so on the basis that the constitution objection relies on a kind of physicalism that is ‘counter-intuitive and no less conceptually problematic than dualism is alleged to be’ since it is almost ‘substance dualism in disguise’.

It is worth pausing to consider the justice of Cooper’s claim that the constitution view is ‘no less conceptually problematic’ than dualism is alleged to be. On the one hand, there are well known problems both for constitution relations in general and for the thesis that persons are constituted by their bodies in particular (see e.g. Olsen, 2001; Inman, 2018). So if Cooper is saying that the constitution view faces problems comparable in severity to those faced by dualism, he may be right. This will depend on contentious issues about the severity of the relevant problems, which I cannot get into here. On the other hand, Cooper might be taken to imply that, because it is almost ‘substance dualism in disguise’, the constitution view faces *the very same* problems that dualism is alleged to face, so that Christian physicalists can have no principled reason to favour the constitution view over dualism. This criticism would be unfair. For as I have explained, the leading philosophical argument for physicalism is the causal exclusion argument. And it is plausible that the constitution view, as a kind of physicalism, will benefit from the support of the causal exclusion argument. (Kim (2005) has argued that the exclusion problem can be extended to non-reductive physicalist views. But proponents of the constitution view can consistently resist this claim, as most nonreductive physicalists do, and proponents of the constitution view are not obliged to adopt a nonreductive view of higher-level properties.) So it is plausible that the constitution view shares with other kinds of physicalism the principal philosophical advantage that physicalism is supposed to enjoy over dualism. The same is likely to be true of many of the subordinate arguments for physicalism, such as arguments from neural correlations. This gives Christian physicalists what many would regard as a powerful reason to favour the constitution view over dualism. It might be worth adding that the suggestion that physicalism is compatible with personal survival without one’s current body is by no means an ad hoc manoeuvre of Christian physicalists in response the biblical case for an intermediate state. The same proposal is, for example, common-place amongst nonreligious physicalists in the literature on transhumanism (see e.g. Kurzweil, 1999; Sandberg & Bostrom, 2008).

In summary, then, the biblical argument for dualism, as Cooper presents it, is built on the claim that passages like Matthew 10:28 and 2 Corinthians 5:8 imply that persons or their souls will exist without their bodies in an intermediate state between death and resurrection and that this entails a form of dualism. The constitution objection shows that there is a gap in this line of reasoning. The claim that persons or souls will exist without their current bodies does not rule out physicalism. Neither does it undermine the principal advantage accorded to physicalism in recent philosophy. So, it looks like the Cooper’s argument poses no direct threat to Christian physicalism.

## The linguistic objection

The constitution objection to the biblical argument for dualism is important. For it seems to allow physicalists to accommodate exactly that biblical evidence which Cooper regards as pointing most decisively in favour of dualism. In the next section I explain why the constitution objection can nonetheless be resisted on the basis of pragmatic considerations. First, however, I will outline what seems to me a more stubborn, linguistic objection. According to this second objection, passages like Matthew 10:28 and 2 Corinthians 5:8 do not just happen to be consistent with physicalism. Rather, they could not in principle contradict physicalism, given the linguistic resources of the Bible.

The linguistic objection rests on an observation about the relationship between concept possession and reference. In order to pick out an entity, *a*, that falls under a concept *F*, you do not need a term that expresses *F*; you just need a term that expresses *some* concept under which *a* falls. On the other hand, in order to refer to all *F*s, actual or possible, or to make claims of the form ‘there are no *F*s’ or ‘*x* exists without any *F*’, you need a term that expresses the concept *F*. (I assume that we individuate concepts by their intensions.) For example, a fifteenth-century Taíno person could refer to the particular admiral, Columbus, as ‘that man’, without having the concept of an admiral. But a fifteenth-century Taíno person could not express the proposition ‘there are no admirals nearby’ or ‘admirals command fleets’, prior to acquiring a term for admirals. In the same way, biblical passages can assert that in the intermediate state, persons or souls will exist without a particular physical thing, like the body. But they cannot straightforwardly assert that persons or souls will exist without any physical thing without a term for physical things.

It follows, I suggest, that even if the constitution objection is set aside, there will remain a gap in the biblical argument for dualism, insofar as it is supposed to show that the Bible implies dualism in the sense that contradicts physicalism. The argument must move from textual evidence that persons or their souls exist without their bodies in the intermediate state to the conclusion that the intermediate state is a nonphysical state. The constitution objection points out that the fact that persons or souls exist without their current bodies does not entail that they exist without *any* body. But suppose that the Bible said that persons or souls will exist without *any body at all* in the intermediate state. Would that show that the intermediate state is a nonphysical state? No. The fact that persons or their souls will exist without anything in the extension of the biblical term being translated ‘body’ (typically ‘σῶμα’ in the New Testament) entails that they will exist without some physical things, but it does not entail that they will exist without any physical thing. For biblical terms such as ‘σῶμα’ express traditional, pretheoretical concepts whose extension is ordinarily significantly narrower than that of the term ‘physical’ in the sense that defines physicalism. For the term ‘physical’ as it defines physicalism has been introduced, in its current usage, specifically in order to accommodate those things posited by modern physics that do not fall under traditional concepts of body or matter. Hence, the biblical argument will leave open the possibility that the person or soul that continues to exist in the intermediate state is or is constituted by a *nonbodily* (i.e. ἀσώματος) physical thing in the sense of ‘body’ used in passages like Matthew 10:28 and 2 Corinthians 5:8. (As I mention below, it *may* be that the biblical authors would have recognised an extended sense of ‘σῶμα’ in which any physical thing that is or constitutes a person could be called their ‘σῶμα’. If so then perhaps we could use this fact to push back against the linguistic objection. But before doing so we would need some reason for thinking that it is this extended use and not a comparatively narrow use that features in passages like Matthew 10:28 and 2 Corinthians 5:8.)

The same point, it seems, will apply to *any* biblical passage that is reputed to threaten Christian physicalism. For the Bible was written in Classical Hebrew, Old Aramaic, and Koine Greek. These languages contain terms for things that are bodily or material, in a pre-theoretical or traditional sense. But they do not contain any term that has the same intension as ‘physical’ as it is used in present-day definitions of ‘physicalism’ and ‘dualism’. Without such a term, it is not obvious how the Bible could articulate a commitment to dualism in the sense that contradicts physicalism. There are two possibilities, neither of which offers much hope. First, a biblical passage might express a commitment to dualism by using a term whose intension is not identical to but subsumes that of ‘physical’. (The intension of one term is subsumed by the intension of a second term if and only if, necessarily, anything in the extension of the first term is also in the extension of the second.) Something that exists without anything in the extension of such a term a fortiori exists in the absence of anything physical. The problem here is that the intension of this term would also have to be narrow enough that it does not include the candidate nonphysical thing (i.e. the soul or person). Biblical languages contain terms that trivially satisfy the first criterion, such as the Greek term for ‘everything’ (πᾶν), but there are no plausible examples that also satisfy the second. Alternatively, it might be possible to construct a complex combination of Hebrew, Aramaic, and/or Greek words that has the same intension, or that subsumes the intension of the modern term ‘physical’. Whether this is in principle possible is too complicated an issue to examine here. (If we knew that the soul where nonphysical, something like ‘τά ἐκτός τῆς ψυχής’, meaning ‘everything other than the soul’ might do. But in the present context this is clearly question-begging.) But even if it is possible, it would be astonishing to find such a construction in a text that is not explicitly engaged in twentieth/twenty-first century analytical metaphysics. It seems then, that there is a gap in the biblical argument for dualism that remains even when the constitution objection has been set aside.

A merit of the linguistic objection is that it formalises the intuitive concern that there is something misguided about looking to the Bible for guidance about a distinctively modern view like physicalism. Ultimately, I think that this concern might be mistaken, and that we might be justified in looking to ancient texts for views about this topic after all. But the idea that such efforts are hopelessly anachronistic is prima facie plausible and deserves serious consideration. Although the linguistic objection is distinct from the constitution objection, it is natural to combine the linguistic objection with a version of the constitution view about relationship between human persons and their bodies. For the most plausible theory that allows human persons to exist as nonbodily physical things in the intermediate state is likely to be one on which we are contingently constituted by our bodies prior to death. Having said this, it is also possible to formulate physicalist theories that can take advantage of the linguistic objection without adopting a version of the constitution view. For example, one can imagine a kind of physicalist soul-body dualism on which human persons (or their ‘souls’) are from the outset identical to nonbodily physical systems which are distinct from and can exist without their organic bodies. (I discuss in section seven what kind of physical systems will count as ‘nonbodily’ in the requisite sense. This depends on how broadly we interpret terms such as ‘σῶμα’in passages like Matthew 10:28 and 2 Corinthians 5:8.) The linguistic objection also differs from the constitution objection in that, whereas the constitution objection relies on the specific content of passages like Matthew 10:28 and 2 Corinthians 5:8 the linguistic objection relies solely on the languages in which the Bible was written. Hence, the linguistic objection promises to free the debate about Christian physicalism from the details of biblical scholarship.

Attempts to locate the philosophical anthropology of the Bible within the categories of recent metaphysics frequently focus on differences between the meanings of biblical terms traditionally translated as ‘soul’ or ‘spirit’, and the present-day meanings of these terms. Comparatively little attention is given to the difference between the meanings of biblical terms translated ‘body’, ‘flesh’ etc. and the present-day philosophical meaning of ‘physical’. But if the argument of this section is successful then this second difference yields a powerful argument that the Bible cannot affirm dualism in the sense that contradicts physicalism.

## A pragmatic response to the constitution objection

Should Christian dualists give up on the biblical argument as an argument against Christian physicalism? Perhaps not. For the linguistic objection suggests a response that has yet to be examined. It is true that the literal meaning of biblical passages is unlikely to entail dualism in the sense that contradicts physicalism. But as linguistics has long recognised, meaning is not exhausted by literal (or ‘semantic’) meaning. There is also pragmatic meaning. So it remains possible to argue that while a literal reading of the passages identified by Cooper does not contradict physicalism, a pragmatically competent reading does. In this section and the next, I sketch what I think is the most promising pragmatic defence of the biblical argument for dualism, focussing on the central pragmatic phenomenon of conversational implicature.

According to a common characterisation, conversational implicature occurs when a speaker means one thing by saying something else. It was named by Paul Grice (1989) who gives the following example:

*A*: I am out of petrol

*B*: There is a garage round the corner.

(Grice, 1989, 32)

Speaker *B* in this conversation has not said that, as far as she knows, the garage is open and has petrol to sell. And yet she clearly intends *A* to infer that, as far as she knows, this is so, and to recognise that this is her intention. In the terminology introduced by Grice, *B*’s utterance *implicates* that as far as she is aware, the garage is open and has petrol to sell.

Grice also advances a theory of conversational implicature that has been very influential. Conversation is necessarily a cooperative enterprise. For this reason, Grice suggests, participants can be presumed to conform to a ‘cooperative principle’, according to which you must make your contribution ‘such as is required, at the stage at which it occurs, by the accepted purpose or direction of the talk exchange in which you are engaged’ (Grice, 1989, 26). Grice supplements the cooperative principle with several ‘maxims’ which specify that you should give neither insufficient nor excessive information, and that you should make your contribution true, relevant, and perspicacious. According to Grice’s theory, conversational implicature occurs when the presumption that an utterance conforms to the cooperative principle and its maxims requires the hearer to infer an implicit meaning. For example, if *B* believed that the garage is closed or out of petrol, her utterance would be irrelevant to *A*, and would therefore violate the cooperative principle. So, the presumption that *B* is not violating the cooperative principle requires the hearer to infer the implicature that as far as *B* knows, the garage is open and has petrol to sell.

More recent works dispute the precise nature of the expectations that generate conversational implicature. Some, like Stephen Levinson (1983, 1987, 2000) stay fairly close to Grice’s cooperative principle and maxims. Others like Deidre Wilson and Dan Sperber (1986, 2012) defend more revisionary views. (Ernst-August Gutt’s (1991) application of Wilson and Sperber’s ‘relevance theory’ has had an immense influence on Bible translation.) Christopher Potts (2015) gives an overview. Nonetheless it is highly plausible that something in the region of the expectations identified by Grice and his followers generates conversational implicatures. And so, if it is plausible from a broadly Gricean perspective that passages like Matthew 10:28 and 2 Corinthians 5:8 implicate dualism, then there is a reasonable chance that they will also do so on whatever theory of conversational implicature we ultimately favour.

In what follows, I outline two broadly Gricean defences of the biblical argument for dualism. The first only gets us part of the way there. It calls into question the constitution objection as Baker presents it, but not the linguistic objection. The second has the potential to rebut the linguistic objection too, but it is based on premisses that are likely to be more controversial. Both attempts to defend the biblical argument assume that the passages identified by Cooper entail that persons or souls continue to exist without their current bodies between death and resurrection. The question is whether, in doing so, they imply a form of dualism, and if so whether it is dualism of the kind that contradicts physicalism.

The first Gricean argument is specifically aimed at rebutting the claim that the passages identified by Cooper can be accommodated by the constitution objection as presented by Baker. The essence of Baker’s response is captured by the following passage:

The Constitution View does not allow that we can exist unembodied; it does allow that we can exist without the bodies that constitute us now… [T]here is no reason to think that the intermediate state must be a disembodied state. For all that we know, persons in the intermediate state are constituted by intermediate-state bodies. So, Cooper’s arguments provide no reason to prefer Mind-Body Dualism. (2004, 11)

But is a pragmatically informed reading of passages like Matthew 10:28 and 2 Corinthians 5:8 consistent with the claim that we—or our souls—can exist without the bodies that constitute us now, *only by obtaining another body*? Supposing that by ‘body’ we mean ‘body’ in the sense that appears in these passages, and not in some distinctively modern sense, the answer is plausibly no.

To see this, imagine a travel agency in New York that advertises an opportunity to ‘get away from the city’. Suppose that the destination of the vacation advertised is in fact Berlin. We would consider this misleading. Why? Plausibly this is because the literal meaning of the expression ‘get away from the city’ is ambiguous. ‘The city’ could denote the particular city you are in, or it could denote the type city, of which all cities are tokens. But if the advert only meant the token city, New York, then it would be violating the cooperative principle. For, it would have been possible to increase the informativeness and perspicacity of the advertisement without cost by referring to New York City by name, or by using a demonstrative pronoun. The advertisement would then have promoted the opportunity to ‘get away from *this* city’ or to ‘get away from *New York*’. The informativeness and perspicacity of the advertisement have not been increased in this way. And so, on the assumption that the advertisement conforms to the cooperative principle, it implicates that the vacation is an opportunity to get away from any token of the type *city*, Berlin included.

We need some story, in any case, about why the description ‘away from the city’ implicates being away from *any* city. And whatever story we favour is likely to apply mutatis mutandis to 2 Corinthians 5:8 where Paul says that we would rather be away from the body (ἐκ τοῦ σώματος) and at home with the Lord. If so, then 2 Corinthians 5:8 implicates that, not only will we exist without our current bodies in the intermediate state, but we will not come into possession of replacement bodies either, at least in the sense of ‘body’ Paul is using in this passage. This seems like a natural reading. After all, if Paul only meant that we will be away from our current bodies then he could easily have increased the informativeness and perspicacity of his claim by inserting a suitable adjective or demonstrative pronoun. For example, he might have said that we would rather be away from *this* body (ἐκ τοῦ σώματος τούτου) (cf. Cooper, 2018, 538). The same line of reasoning can be applied to Matthew 10:28 and to 1 Philippians 20:24 which resembles 2 Corinthians 5:8 except that it employs ‘σάρξ’ (usually translated ‘flesh’) rather than ‘σῶμα’. In each case, the literal content of the passage is compatible with the thesis that in the intermediate state persons or souls only exist without their *current* bodies, but a pragmatically informed reading seems to suggest that in the intermediate state persons or souls exist without *any* body.

At face value then, several of the passages that Cooper identifies implicate that persons or souls will exist without *any* body in the intermediate state. Proponents of the constitution objection can, of course, argue that countervailing factors undermine the apparent implicature. But it is not clear what factors could serve this purpose. For while it is easy to marshal evidence against interpretations that entail an intermediate state *of any sort*—for example, the fact that the intermediate state seems to call into question the purpose of the resurrection—it is much harder to identify evidence against interpretations that entail a *nonbodily* intermediate state only. It is evidence of the second kind that is needed, if the constitution objection is to serve its purpose in parrying the biblical argument for dualism while allowing for the possibility of an intermediate state. On the other hand, the wider contexts discussed by Cooper tend to reinforce the apparent implicature. For example, Cooper argues that the idea of a disembodied intermediate state was commonplace in intertestamental Judaism, just as the idea of a disembodied afterlife was in the surrounding Greek culture. It follows that the authors of texts like Matthew 10:28 and 2 Corinthians 5:8 had good reason to expect that their audiences would hear these passages as affirmations of a disembodied intermediate state. If so, the thesis that our understanding of these passages should be restricted to their literal content seems still more difficult to square with the cooperative principle.

Pending some response from proponents of the constitution objection, it seems reasonable to conclude that *if* we read passages like Matthew 10:28 and 2 Corinthians 5:8 as affirming the existence of an intermediate state *then* we should read them as affirming the existence of a disembodied intermediate state, in the sense of ‘body’ used in these passages. Baker (2011, 55) says ‘I know of no reason—Biblical or philosophical—to suppose that the intermediate state must be a disembodied state.’ The argument of this section provides one such reason. If it is successful, then the constitution objection does not afford Christian physicalists a satisfactory response to the biblical argument for dualism after all.

This first Gricean argument does important work. If it is successful, and if passages like Matthew 10:28 and 2 Corinthians 5:8 affirm the existence of an intermediate state at all, then it seems to follow that they also presuppose a *kind* of dualism in the sense that they draw a distinction between souls or persons and bodies, and affirm that souls or persons can exist without bodies. However, it does not follow that the Bible implies dualism in the sense that contradicts physicalism. The apparent implicature of passages like Matthew 10:28 and 2 Corinthians 5:8 is that in the intermediate state, persons or souls will exist without bodies in the traditional or pretheoretical sense of ‘body’ used in those passages. This probably includes all organic bodies. Perhaps it includes material objects more broadly. But it almost certainly excludes many things that fall in the extension of the term ‘physical’ as it is used to define physicalism. So, the argument of this section leaves open the possibility that while the intermediate state is a nonbodily state in the sense of ‘body’ used in passages like Matthew 10:28 and 2 Corinthians 5:8, it is nonetheless a physical state. Furthermore, although the constitution objection to the biblical argument for dualism appears to be unsatisfactory, relying as it does on the idea that passages like Matthew 10:28 and 2 Corinthians 5:8 only imply that persons or souls exist without their *current* bodies after death, this does not mean that we must give up on the constitution view about the relationship between human persons and their bodies on which that objection depends. For although Baker and Corcoran do not stress this point, the constitution view as they present it does not say that human persons are necessarily constituted by bodies in a traditional or pretheoretical sense of ‘body’, but that they are necessarily constituted by bodies in the modern sense of the kind of things described by physics (see e.g. Baker, 2011, 48; Corcoran 2018, 374). Insofar as this is so, proponents of the constitution view can take advantage of the linguistic objection.

## A pragmatic response to the linguistic objection?

The argument of the foregoing section gives us an additional reason to regard the linguistic objection to the biblical argument for dualism is valuable. The constitution objection says that because passages like Matthew 10:28 and 2 Corinthians 5:8 only entail that persons or souls can exist without their *current* bodies after death, it remains possible that they do so only by acquiring replacement bodies. It is plausible that the implicatures of passages like Matthew 10:28 and 2 Corinthians 5:8 do not permit this, even if their literal content does. The linguistic objection is more stubborn. It says that even if passages like Matthew 10:28 and 2 Corinthians 5:8 implicate that persons or souls will exist without *any* body at all, in the sense of ‘body’ used in these passages, it will remain open that they will nonetheless be constituted by or simply identical to, some *nonbodily physical thing*. It is not so easy to argue that the implicatures of the passages identified by Cooper rule *this* out. For those passages do not use the concept of physical things, if only because no term in the biblical languages expresses that concept.

Should Christian physicalists be content to rest their response to the biblical argument for dualism on the linguistic objection? This depends on how much weight one accords different kinds of argument in favour of physicalism. Those motivated by theological arguments may see limited value in maintaining a form of Christian physicalism that concedes the possibility of a disembodied intermediate state. For the theological motivations for Christian physicalism are often tied to traditional or pretheoretical concepts of *body* and *flesh—*concepts that have great importance in Christianity. If the argument of the last section is successful then Christian physicalists whose principal concern is to maintain that human persons are essentially bodily might do better to resist outright the claim that the Bible implies that persons or souls continue to exist in an intermediate state after death, rather than attempt to accommodate this possibility on the basis that we might acquire replacement bodies. By contrast, those motivated primarily by philosophical arguments are likely to see significant value in maintaining Christian physicalism, even while conceding the possibility of a disembodied intermediate state. For the philosophical motivations for physicalism tend to be tied to the modern concept of physical things, understood as those things described by physics. For example, so long as it is compatible with physicalism, it is probable that the philosophical anthropology implied by the Bible will be immune to the casual exclusion argument and, more broadly, to the charge that it is incompatible with the findings of physical science.

It might also be possible to maintain that the intermediate state is a bodily state in *some* biblical sense of ‘body’ while conceding that it is a nonbodily state in the sense that appears in passages like Matthew 10:28 and 2 Corinthians 5:8.^1^ (Compare: Democritus draws a sharp distinction between the body (usually ‘σῶμα’) and the soul (‘ψυχή’) (Diels and Kranz 1951–2, 68B 31, 37, 159, 187), but holds that the soul is composed of atoms of fire, and is in this sense itself a body, albeit of an ‘especially nonbodily’ (‘μάλιστα ἀσώματον’) sort (Diels and Kranz 1951–2, 67A 28, 68A 101, 104a).) How plausible this is depends on how broad we think the use of ‘body’ in passages like Matthew 10:28 and 2 Corinthians 2:8 is. One piece of evidence that is relevant here is 1 Corinthians 15:44 where Paul says that the resurrection body will be a ‘σῶμα πνευματικόν’, usually translated ‘spiritual body’. This could be taken in two ways. On the one hand it might be taken as evidence for a comparatively broad use of ‘body’, which might apply to the intermediate state, even if the sense of ‘body’ used at Matthew 10:28 and 2 Corinthians 2:8 does not. Alternatively, we might reason that since 2 Corinthians 5:8 contrasts being ‘away from the body’ with the resurrection, here too ‘body’ must be used in a comparatively broad sense that would include the ‘σῶμα πνευματικόν’ of the resurrection. In that case, the intermediate state will have to be a nonbodily state even in this broad sense of ‘body’, and the prospects for finding a biblical use of ‘body’ broader than that of 2 Corinthians 5:8 will be that much slimmer. Even if we cannot find biblical evidence for a broader sense of ‘body’ than that of passages like Matthew 10:28 and 2 Corinthians 5:8, we might reason that there is an intuitive use of the English natural-language concept ‘body’ that applies to *any* physical thing that is or constitutes a person, and that the biblical authors might have recognised such a use of ‘σῶμα’. (To my mind it is plausible that any fundamentally nonmental thing that is or constitutes a person could be thought of as their ‘σῶμα’ in an extended or analogical sense.) This issue deserves to be considered further elsewhere. For now, suffice it to say that those who rely on the linguistic objection to the biblical argument for dualism must concede that the intermediate state is a disembodied state in *one* biblical sense of ‘body’, but not necessarily that it is a disembodied state in *every* biblical sense of ‘body’. Christian physicalists may take some comfort in this.

At least some Christian physicalists, then, will have good reason to press the linguistic objection to the biblical argument for dualism, even if it has been conceded that a pragmatically informed reading of passages like Matthew 10:28 and 2 Corinthians 5:8 suggests that there is a disembodied intermediate state, in an important sense of ‘disembodied’. Christian physicalists who adopt this position will say that human persons or souls are necessarily physical but that they can continue to exist after death as physical things that are not bodies in the sense of ‘body’ used in passages like Matthew 10:28 and 2 Corinthians 5:8 is. What would such an existence look like? This depends on what conditions we think are necessary for the continued existence of persons or souls, and (again) on how broad we take the use of ‘body’ in passages like Matthew 10:28 and 2 Corinthians 5:8 to be. We may take inspiration from the literature on transhumanism. A common transhumanist strategy for explaining how we might survive in the absence of bodies, in the sense of human organisms, appeals to two widely accepted theories about human nature: computationalism about psychological states and the psychological continuity theory about personal identity (see e.g. Kurzweil, 1999, 383; Weir, 2018). According to these theories, a person continues to exist so long as their psychological states continue (in accordance with a suitably demanding continuity relation) and a person’s psychological states continue so long as the underlying computational processes are maintained. On this basis transhumanists have argued that human persons could survive death as computations implemented on ‘utility fog’, a substance composed of nanorobots. Suppose that the term translated as ‘body’ in passages like Matthew 10:28 and 2 Corinthians 5:8 has a comparatively narrow meaning, so that only applies to e.g. organic bodies or more or less solid, unitary objects. (We might think here of the sense of ‘σῶμα’ used by Democritus when he contrasts the soul with the body, even though he regard the soul as itself a body in a broader sense of the term.) In that case existence as a computation implemented on ‘utility fog’ is likely to count as disembodied physical existence in the requisite sense. So too, for that matter, is survival as the cloud of fiery atoms with which the ancient atomists identified the soul.

It may be that ‘body’ in passages like Matthew 10:28 and 2 Corinthians 5:8 has a broader extension than one that includes only organic bodies and more or less unitary objects. As we have just seen, the context of 2 Corinthians 5:8 might be taken to indicate that ‘σῶμα’ here has a broad sense that will include Paul’s ‘σῶμα πνευματικόν’. In that it case it seems likely that ‘bodies’ will also include the utility fog of the transhumanists and the nebulous soul of the atomists. (In a phrase similar to 1 Corinthians 15:44, Epicurus (Diogenes Laertius, Lives, 10.63) calls the soul on this view a body ‘most resembling breath’ (‘προσεμφερέστατον δὲ πνεύματι’).) But it is plausible that modern physics supplies entities that will not count as bodies even in this comparatively broad sense of ‘body’. For our most complete theories of elementary ‘particles’ represent them not as small solid billiard ball-like objects, but as (very oddly behaved) excitations in the fundamental physical fields. The intuitively body-like entities of classical physics, even those that resemble breath or fire, only emerge when you have large numbers of these excitations occurring together in the right combinations. If computationalism and the psychological continuity theory are true then there is no reason why God should not implement the computational processes that are sufficient for your continued existence as, for example, a pattern of excitations in the electron field. Indeed, doing so might be more efficient than implementing them in a classical system, given the advantages of quantum computing. In this scenario, you will exist without anything that Democritus, Epicurus or Hobbes would have recognised as a ‘body’, and it is plausible that the same will go for the sense of ‘body’ that features in passages like Matthew 10:28 and 2 Corinthians 5:8, even on a broad interpretation of its meaning.

It is plausible, then, that even when it is reinforced by pragmatic considerations, the biblical argument for dualism will at most show that passages like Matthew 10:28 and 2 Corinthians 5:8 imply that persons or souls will exist without bodies in the intermediate state, in a traditional or pretheoretical sense of ‘body’, not that they will do so without anything physical. If so, then Christian dualists must rely on other arguments to make the case for dualism in the sense that contradicts physicalism. There exist two ways proponents of the biblical argument for dualism might push back at this stage. First, as I mentioned in section three, they might argue that in fact, the sense of ‘body’ used in passages like Matthew 10:28 and 2 Corinthians 5:8 is *so broad* that it will apply to any physical realization of a person or soul—even to a pattern of excitations in the electron field. Secondly, they might argue that those physical theories that permit personal survival without bodies in the sense of ‘body’ used in passages like Matthew 10:28 and 2 Corinthians 5:8 are physically or metaphysically implausible. Both responses deserve consideration, but I will not discuss them here. Instead, I will conclude by considering what seems to me a more promising argument for the thesis that passages like Matthew 10:28 and 2 Corinthians 5:8 imply dualism in the sense that rules out physicalism. Although I think this argument promising, I also have some doubts about it in view of which I think it is likely to have limited dialectical force, at least pending further investigation. The argument I have in mind is based on the thought that, although there are physicalist views that permit persons or souls to exist without bodies after death, these views are comparatively unnatural, whereas dualism of the sort that rules out physicalism, though it may be false, is very natural. It follows that when someone affirms that persons or souls will exist without bodies after death, it is probable that they are presupposing dualism of the sort that rules out physicalism. And if they do nothing to forestall this assumption, there will be a case for thinking that they thereby implicate dualism of the kind in question. Hence there will be a case for thinking that if passages like Matthew 10:28 and 2 Corinthians 5:8 implicate that the intermediate state is a disembodied state then they also implicate that it is a nonphysical state.

This argument rests, not only on the Gricean notion of implicature, but also on the claim that there is something peculiarly natural about dualism of the sort that rules out physicalism. This claim can be supported by the widely affirmed thesis that humans are naturally drawn to a ‘common-sense’ or ‘folk’ dualist picture of human nature that resembles traditional substance dualism. K. Mitch Hodge (2008, 388) lists multiple researchers in philosophy, psychology, anthropology and biology who affirm something in the vicinity of this claim. The most vocal among them is the psychologist Paul Bloom (2004, 2007). Bloom asserts that from early infancy humans instinctively draw a sharp distinction between material bodies, including the human body, and immaterial minds or souls. He describes the resulting picture of human nature as ‘a strong substance dualism of the sort defended by philosophers like Plato and Descartes’ (Bloom, 2007, 149). Proponents of the thesis that humans are naturally drawn to a common-sense form of dualism tend to take it for granted that the sort of dualism in question is incompatible with physicalism. However, this requires explanation. For young infants and historical philosophers like Plato and Descartes are no more acquainted with modern physics than the authors of the Bible. And so if the dualist position that they favour is incompatible with physicalism, this cannot be because they believe that minds or souls are not among the things described by modern physics. If it is incompatible with physicalism this is presumably because, although common-sense dualism does not involve the idea that minds or souls are left out by physics, it does involve the idea that minds or souls have some characteristic *F* where, as a matter of fact, nothing described by physics is *F*. (That is, common-sense dualism will include a content such as < minds or souls are *F* > where in fact nothing described by physics is *F*. The content of common-sense dualism will not itself concern modern physics.) For example, common-sense dualism is associated with the idea that minds or souls are publicly unobservable. It is plausible that everything described by physics is publicly observable, either directly or via instruments. If so, then it follows that the minds or souls of common-sense dualism are not physical in the sense that defines physicalism.

There are multiple plausible candidates for *F*, and so it seems probable that one or more will do the job. In view of recent discussions of the definition of physicalism, a particularly salient candidate for *F* is the property of being fundamentally mental. Minds or souls are fundamentally mental if and only if they do not exist in virtue of more basic nonmental things. For example, Cartesian souls cannot be analysed or disaggregated into more basic nonmental parts or properties, roughly because Cartesian souls have no proper parts and all of their properties are ‘modes’ of the ‘principal attribute’ thought. In this respect, Cartesian souls differ markedly from physical things as we usually conceive of them, whether they are human bodies, clouds of nanorobots, or patterns in the fundamental physical fields. Bloom claims that the common-sense dualist picture which humans naturally favour closely resembles that of Descartes. If so, then presumably common-sense dualism also represents minds or souls as fundamentally mental. It is, moreover, independently plausible that, insofar as there exist psychological factors that make us natural dualists, they do so in part because they make it natural to think of our mental lives as fundamentally mental. If common-sense dualism represents minds or souls as fundamentally mental then this gives us a particularly straightforward reason for thinking that common-sense dualism is incompatible with physicalism. For, not only is it widely believed that no physical thing is in fact fundamentally mental, but also, most philosophers are happy to stipulate that this must be so if physicalism obtains. (Again, see Wilson (2006, fn. 1) for examples.) This stipulation is useful in responding to Hempel’s dilemma and in explaining why physicalism is incompatible with Russellian forms of panpsychism.

There is, then, a case for thinking that humans are naturally drawn to a common-sense dualist picture of human nature that rules out physicalism. Suppose, which seems likely, that there is no comparably natural view that allows persons or souls to exist without bodies in a traditional or pretheoretical sense of ‘body’ and that is compatible with physicalism. It follows that when someone affirms that persons or souls will exist without bodies after death that it is, all things being equal*,* probable that they are presupposing dualism of the sort that rules out physicalism. The final step in our argument says that, for this reason, if such a speaker does nothing to indicate otherwise, dualism of the sort that rules out physicalism is part of the implicature of their utterance. This is plausible because the speaker in such a scenario is in a position to expect their audience to assume that they are presupposing the common-sense dualist picture of human nature to which we are all drawn. And so, supposing that they are conforming to the cooperative principle, they should indicate if this is not so. (It is necessary to assume here that implicatures need only follow abductively, and not deductively, from the utterance and the assumption that the speaker conforms to the cooperative principle, but this assumption is widely accepted (cf. Rysiew, 2000, 577).) The implicature will be stronger the more familiar the speaker and audience are with the common-sense dualist picture it involves. For this reason, the line of argument proposed here might be reinforced by contextual factors. For example, if it is true that humans are naturally drawn to a common-sense dualist view that rules out physicalism, then it seems likely that the authors and original audience of the Bible would have been familiar with manifestations of this view in Platonic, Pharisaic, and folk conceptions of the soul-body relation. If so, this will increase the probability that the original audience of the Bible would have understood passages like Matthew 10:28 and 2 Corinthians 5:8 as endorsements of the common-sense dualist view in question. This, in turn, will increase the severity of the breach in the cooperative principle if the authors are not endorsing such a position. The strength of the implicature will also be greater the greater the degree naturalness that we assign to common-sense dualism in comparison with physicalist views that permit persons or souls to exist without bodies in the sense of ‘body’ used in passages like Matthew 10:28 and 2 Corinthians 5:8.

There is, therefore, a pragmatic case for thinking that if passages like Matthew 10:28 and 2 Corinthians 5:8 implicate that the intermediate state is a disembodied state, then they also implicate that it is a nonphysical state. As I have indicated, I think this argument should be taken seriously. The literature on common-sense dualism has not yet, to my knowledge, investigated the role of the psychological factors underlying common-sense dualism in generating conversational implicatures. This is likely to be a fruitful line of enquiry which might shed light on, and to some extent justify, the impulse to read passages like Matthew 10:28 and 2 Corinthians 5:8 as endorsements of dualism of the sort that rules out physicalism. For two reasons, however, I doubt that the argument sketched here can yield a dialectically powerful case for thinking that the Bible implicates dualism in the sense that contradicts physicalism without further reinforcement. These reasons concern, respectively, the claim that humans are naturally drawn to a form of common-sense dualism of the sort that contradicts physicalism and the claim that if so then passages like Matthew 10:28 and 2 Corinthians 5:8 implicate dualism of the kind in question.

The argument I have outlined rests on the assumption that humans are naturally drawn to a form of common-sense dualism that resembles traditional substance dualism. If the argument is to succeed, it is crucial that that the common-sense dualist picture in question should represent minds or souls as having some characteristic *F*, such as that of being fundamentally mental, where no physical thing is *F*. Although this assumption is plausible, it is far from certain. Hodge (2008, 2018, 2021) has identified a number of cultural, philosophical and theoretical problems for Bloom’s claim that humans are natural Cartesians, and has developed an alternative account of the data reputed to support this hypothesis. Edward Slingerland and Maciej Chudek (2011, 998) report that a quantitative study of mind and body concepts in ancient China supports Hodge’s position over Bloom’s. A particularly clear problem for the claim that the cognitive default resembles Descartes’s dualism is the fact that many putative expressions of folk dualism represent disembodied souls as perceptible entities, whose disembodiment is evinced by their transparency, diffuseness, or peculiar behaviours such as appearing and vanishing at will. This way of representing disembodiment is as old as the Odyssey and the Epic of Gilgamesh, and turns up in biblical passages such as Matthew 14:26 and Luke 24:37 when the disciples suppose Jesus to be a ‘φάντασμά’ or ‘πνεῦμα’, both typically translated ‘ghost’. This way of representing disembodiment coheres poorly with Descartes’ dualism, and does not obviously imply that souls are nonphysical entities, either because they are fundamentally mental or for some other reason. It is true that other evidence, such as the tendency of infants to assume that our psychological lives continue after death, is more favourable to the view that the common-sense dualist position resembles that of Descartes (cf. Bering, 2006). But pending further research any argument that rests on the assumption that this is so will be open to serious challenges. Michael Antony (2006) proposes some lines of enquiry which would be particularly helpful in determining in what respects the cognitive default resembles Descartes’ dualism. The recently inaugurated research programme on the ‘meta-problem of consciousness’ is also highly relevant (cf. Chalmers, 2018; Kammerer 2020). This is the problem of explaining why we think that there is a problem about the idea that consciousness is physical in the first place. It is relevant because a popular hypothesis is that there exist deep-seated and widespread features of human psychology that drive us to think of our mental lives in a way that rules out physicalism. If so then explanatory parsimony suggests that these same factors play an important role in accounting for common-sense dualism and that we should, therefore, expect common-sense dualism to be dualism of the sort that rules out physicalism.

The argument of this section also depends on the thesis that if humans are naturally drawn to a form of common-sense dualism that contradicts physicalism, then passages like Matthew 10:28 and 2 Corinthians 5:8 implicate dualism of this kind. I have suggested that this is plausible on the basis that these passages do seem to implicate that persons or souls exist without bodies in the intermediate state. For this reason, if it is true that humans are naturally drawn to a form of common-sense dualism that contradicts physicalism then there seems to be a cogent abductive argument from the content of passages like Matthew 10:28 and 2 Corinthians 5:8, and the assumption that the authors are adhering to Grice’s cooperative principle, to the conclusion that the authors are presupposing common-sense dualism of the sort in question. I have also suggested that the implicature might be reinforced by contextual factors. However, even granting these claims, the presence of the implicature can be challenged. One reason for this is that implicatures depend on the relevance of their content in the context in which they occur. In Grice’s terms, they depend on the ‘accepted purpose or direction of the talk exchange’. There is therefore room for Christian physicalists to argue that because the New Testament is not a manual of metaphysics its audience have no reason to suppose that passages like Matthew 10:28 and 2 Corinthians 5:8 will communicate an accurate account of the ontology of human persons. (Murphy (2006, 21) suggests, in the vein, that the Bible’s ambiguity on questions of philosophical anthropology indicates that it does not affirm any doctrine on this issue.) For this reason, even if it is true that passages like Matthew 10:28 and 2 Corinthians 5:8 would implicate dualism of the sort that rules out physicalism if they occurred in contexts where philosophical anthropology is at issue, it is not obvious that they do so in their actual context.

Neither of these two lines of objection strikes me as decisive. On balance, my judgement is that the data on common-sense dualism probably is best explained by the thesis that we have a natural tendency to think in terms of something like Descartes’s substance dualism, and that the passages identified by Cooper may well implicate dualism of the kind in question. But I would be hesitant to place significant weight on the argument outlined in this section without further evidence that humans are strongly drawn to a form of common-sense of the sort that rules out physicalism and that readers of the Bible can reasonably expect passages like Matthew 10:28 and 2 Corinthians 5:8 to communicate what the authors take to be an accurate understanding of the ontology of human persons. Evidence of the first kind may be sought in future research on common-sense dualism and related issues such as the meta-problem of consciousness. These research programmes are relatively young and it is possible that the requisite evidence will be forthcoming. Evidence of the second kind is likely be affected by all sorts of abstruse hermeneutical issues and for this reason, I am less confident about the prospects for progress in this regard.

In view of the concerns raised, it seems likely that the linguistic objection will render the biblical argument for dualism of limited dialectical force as an argument against Christian physicalism, even when it has been accepted that passages like Matthew 10:28 and 2 Corinthians 5:8 implicate a disembodied intermediate state. If so, Christian physicalists who are open to the possibility of an intermediate state can rely on the linguistic objection to the biblical argument for dualism, so long as they are willing to concede that the intermediate state is not a bodily state, in the sense of ‘body’ expressed by biblical terms like ‘σῶμα’ and ‘σάρξ’ as they are used in the relevant passages. Those Christian physicalists who find this unacceptable should either seek a response to the Gricean argument of the previous section or resist the idea that the Bible implies the existence of an intermediate state of any kind. Christian dualists might benefit from examining how to improve the Gricean response to the linguistic objection outlined in this section, taking into account new research on common-sense dualism and the meta-problem of consciousness. Christian dualists who see the linguistic objection as successful should rely on other arguments to establish dualism in the sense that contradicts physicalism.

## Footnotes

1 I am grateful to an anonymous reviewer for the International Journal of Philosophy of Religion for the points raised in this paragraph.
